# Diagnostic Accuracy of Two Multimodal Artificial Intelligence Systems Versus Dental Students in Endodontic Radiographic Classification

**DOI:** 10.3390/dj14100679

**Published:** 2026-10-19

**Authors:** Andrea Spinelli, Emanuele Charles, Maria Giovanna Gandolfi, Carlo Prati, Fausto Zamparini

**Affiliations:** 1 Endodontic Clinical Section, Dental School, Department of Biomedical and Neuromotor Sciences, University of Bologna, 40125 Bologna, Italy; emanuele.charles@studio.unibo.it (E.C.); carlo.prati@unibo.it (C.P.); fausto.zamparini2@unibo.it (F.Z.); 2 Laboratory of Green Biomaterials and Oral Pathology, Dental School, Department of Biomedical and Neuromotor Sciences, University of Bologna, 40125 Bologna, Italy; mgiovanna.gandolfi@unibo.it

**Keywords:** artificial intelligence, endodontics, periapical radiography, apical periodontitis, diagnostic accuracy, dental education

## Abstract

**Objectives:** Multimodal artificial intelligence (AI) systems are increasingly used to support image interpretation in dentistry, but evidence regarding their diagnostic performance on endodontic radiographs remains limited. This study compared the diagnostic accuracy of two general-purpose multimodal AI systems with that of undergraduate and postgraduate dental students and evaluated whether image cropping influenced AI performance. **Methods:** Fifty anonymized periapical radiographs were retrospectively selected, with 10 cases in each of five predefined endodontic diagnostic categories. Reference diagnoses were established by consensus between two experienced endodontists. ChatGPT version 5.2 and Gemini version 3 independently classified each case using both full-size and tooth-cropped images and a standardized prompt. Twenty-five fifth-year undergraduate dental students and 20 postgraduate students in clinical endodontics classified the full-size images. Overall and category-specific accuracies were calculated, and agreement with the reference standard was assessed. **Results:** Overall accuracy was highest among postgraduate students (84.9%), followed by undergraduate students (78.7%). Gemini achieved accuracies of 72.0% with full-size images and 82.0% with cropped images, whereas ChatGPT achieved 68.0% and 50.0%, respectively. Cropping significantly reduced ChatGPT accuracy (*p* = 0.0117) but did not significantly affect Gemini performance (*p* = 0.3018). Diagnostic accuracy varied substantially across categories. Agreement with the reference standard for the AI systems ranged from 50.0% to 82.0%. **Conclusions:** Diagnostic performance depended on the evaluator, AI system, image format, and diagnostic category. Postgraduate students showed the highest overall accuracy, while AI performance was heterogeneous and did not consistently exceed that of trained human observers. General-purpose multimodal AI may support supervised radiographic interpretation and education, but it should not replace comprehensive clinical assessment or professional judgment.

## 1. Introduction

Artificial intelligence (AI) is increasingly used in dentistry to support image interpretation, diagnostic decision-making, treatment planning, and outcome prediction [1,2,3,4]. Machine-learning and deep-learning methods have been applied to dental radiographs and cone-beam computed tomography for tasks including assessment of root morphology, segmentation of pulp spaces, and detection of periapical radiolucencies [5,6,7,8]. Although dedicated algorithms can perform well within defined tasks, their accuracy depends on the training data, image quality, reference standard, and target population [1,2,7,8].

Radiographic assessment is central to endodontic diagnosis and follow-up, but two-dimensional periapical images may contain signs, superimposed anatomy, and projection-related limitations. Moreover, a definitive pulpal or apical diagnosis normally integrates symptoms, patient history, sensibility testing, percussion and palpation, periodontal probing, and restorability assessment [9,10]. Interpretations and treatment decisions may therefore vary with clinical experience and training [11,12].

General-purpose large multimodal models can process both text and images and are accessible to clinicians and students [4,13,14]. Recent studies have compared such systems with dental students in endodontic case-based questions [15,16] and have examined their performance on visually based dental examinations [17]. A newly published radiograph-only study found that ChatGPT-5 had high specificity but low sensitivity for inadequate root fillings, voids, and periapical pathosis [18]. These findings suggest that performance may depend strongly on the model, task, available clinical context, and image presentation. Evidence specifically comparing more than one general-purpose multimodal AI system with observers at different training levels on standardized periapical radiographs remains limited.

The primary aim was to compare the radiographic classification performance of ChatGPT version 5.2 and Gemini version 3 with that of fifth-year undergraduate dental students and postgraduate students in clinical endodontics using full-size periapical radiographs. The secondary aim was to determine whether image presentation (full-size versus tooth-cropped images) influenced AI performance.

## 2. Materials and Methods

### 2.1. Study Design, Reporting, and Ethical Considerations

This retrospective comparative diagnostic accuracy study evaluated two multimodal AI systems and two groups of human observers. The manuscript was prepared in accordance with the CLAIM 2024 and STARD-AI reporting recommendations [19,20]. Periapical radiographs were retrieved from the digital imaging archive of the Endodontic Clinical Section, University of Bologna, Italy, using XDENT software (CGM XDENT Software S.r.l., version 2026.2, build 0.2, Ragusa, Italy). The pre-existing radiographs were acquired during routine care from patients’ clinical chart. Images were then irreversibly anonymized before they were made available to the investigators; no identifiers, re-identification keys, or other identifiable clinical information was accessed or submitted to the AI systems.

Formal ethics approval was not obtained because the study used exclusively pre-existing, fully anonymized radiographic data and the observer component was anonymous, voluntary and non-interventional.

All observer participants were informed about the study and voluntarily agreed to the anonymous collection and use of their responses for research purposes. No identifiable participant information is reported in this manuscript. The study was conducted in accordance with applicable institutional data-protection requirements and the principles of the Declaration of Helsinki.

### 2.2. Radiographic Case Selection

Investigators screened the archive for radiographs of adequate quality that clearly showed a target tooth and the surrounding periapical tissues, contained sufficient radiographic and clinical documentation to establish a reference diagnosis, and represented one of the predefined diagnostic categories. Selection was purposive rather than random or consecutive, with the specific aim of obtaining five balanced diagnostic groups comprising 10 cases each. Radiographs were excluded when major artefacts, projection errors, distortion, overlapping structures, or incomplete documentation impaired interpretation. A balanced sample of 50 cases was selected, with 10 cases in each category: (1) healthy teeth; (2) carious lesions without radiographic pulp exposure or apical periodontitis; (3) untreated teeth with apical periodontitis compatible with pulp necrosis; (4) root canal-treated teeth without apical periodontitis; and (5) root canal-treated teeth with apical periodontitis.

### 2.3. Reference Standard and Anonymization

The reference diagnosis was established before observer testing by consensus between two academic endodontists with more than 10 years of clinical experience. The experts reviewed the available clinical and radiographic documentation, together with the appearance of the target tooth and periapical tissues. Disagreements were resolved by discussion. An independent operator who had no access to patient information exported the selected images and assembled the assessment files. The reference diagnoses were concealed from all human observers and AI systems.

### 2.4. Image Preparation

Periapical radiographs were acquired using an X-Mind DC intraoral X-ray unit (Acteon, Mérignac, France) in digital mode at 70 kV, 8 mA, and an exposure time of 0.20 s. Images were recorded using size-2 photostimulable phosphor plates (DÜRR DENTAL SE, Bietigheim-Bissingen, Germany) and digitized using a VistaScan Combi View scanner (DÜRR DENTAL SE). According to the manufacturer specifications, the scanning system provides an effective spatial resolution of up to 22 lp/mm, a theoretical resolution of up to 40 lp/mm, a pixel pitch of 12.5 µm, and 16-bit grayscale depth. The original radiographs were exported from XDENT as high-resolution JPEG files. Images were subsequently imported into Microsoft PowerPoint for preparation of the cropped condition, with default resolution set as “High Fidelity” and the “Do not compress images” enabled.

Cropping was performed manually by removing peripheral portions of the image while preserving the complete crown, roots, and periapical region of the target tooth. Image proportions and the aspect ratio were maintained throughout the procedure, without geometric distortion. No contrast adjustment, filtering, sharpening, or other image enhancement was applied. High-resolution image files were subsequently generated for assessment. Human observers evaluated the full-size image set, whereas both AI systems evaluated the full-size and cropped image sets.

### 2.5. Human Observers

The postgraduate group comprised 20 dentists aged 25–29 years who were enrolled in the Master’s programme in Clinical Endodontics and had completed its theoretical component. From February to June 2026, they regularly attended both the Master’s course and the Endodontic Clinical Section from Mondays to Thursdays, where they participated in supervised clinical activities. All participants had previous clinical experience in dentistry and endodontics. The undergraduate group comprised 25 fifth-year dental students enrolled in the Degree Programme in Dentistry at the University of Bologna. Participants had received training in endodontics and periapical radiographic interpretation. Participants received standardized written instructions, were informed about the study, and voluntarily agreed to the anonymous use of their responses; participation did not affect academic evaluation.

Human observers assessed the full-size image set. No symptoms, sensibility-test findings, percussion or palpation findings, periodontal probing, or patient history was provided. Each observer independently classified all 50 target teeth into one of the five predefined categories. Responses were collected using Google Forms, and observers were blinded to the reference diagnoses and to the responses of other participants.

### 2.6. Artificial Intelligence Systems

The evaluated systems were ChatGPT version 5.2 (OpenAI, San Francisco, CA, USA) and Gemini version 3 (Google, Mountain View, CA, USA). Each system independently assessed all 50 cases in both formats. The target tooth was identified using FDI numbering. Each case was analyzed independently, no feedback was provided, and only one final classification was accepted. AI testing was performed in March 2026. ChatGPT was accessed through the ChatGPT Plus web interface, whereas Gemini 3.0 was accessed through the institutional University account. For each AI system, all cases were evaluated within a single session, without initiating a new conversation for each individual case. Each image was submitted once, and the first final classification generated by the model was retained without re-querying or corrective prompting.

A standardized prompt instructed each system to classify the target tooth using radiographic findings only. The requested assessment included coronal integrity, caries depth, previous root canal treatment, periodontal ligament space, lamina dura, periapical radiolucency, alveolar bone crest, and surrounding supporting tissues. The models were explicitly instructed not to suppose unavailable clinical information. When uncertain, they had to select the single most likely category; no additional category was permitted. The complete prompt is provided as Appendix A. Each case was evaluated once, and the first final classification generated by the model was retained. No re-querying, corrective prompting, or post hoc response selection was performed. The predefined endpoint was first-response diagnostic performance under a standardized single-interaction workflow, rather than inter-session reliability or repeated performance.

### 2.7. Outcome Measures and Statistical Analysis

The primary outcome was diagnostic accuracy, defined as the proportion of classifications that agreed with the reference diagnosis. Accuracy was calculated overall and by diagnostic category. AI accuracy was calculated separately for full-size and cropped images; human accuracy was calculated by pooling all responses within each observer group. Secondary outcomes were the effect of image format within each AI system and agreement with the reference standard. Direct human–AI comparisons were restricted to the full-size image condition. Cropped-image results were analyzed exclusively as a within-AI image-format experiment.

No formal a priori sample-size calculation was performed. The study was designed as an exploratory balanced diagnostic benchmark, and 10 radiographs were included in each predefined category to ensure equal representation of the five diagnostic conditions within a feasible observer-assessment workload.

Categorical data were summarized as frequencies and percentages. Human-observer responses were analyzed at the individual-response level using mixed-effects logistic regression, with classification accuracy (correct/incorrect) as the dependent variable. Observer group, diagnostic category, and their interaction were included as fixed effects, with crossed random intercepts for observer and radiographic case to account for repeated evaluations by the same observer and multiple evaluations of the same radiograph. Model-based marginal estimates of accuracy with 95% confidence intervals were obtained for the undergraduate and postgraduate groups.

For the AI systems, accuracy was calculated separately for each model and image format as the proportion of classifications agreeing with the reference diagnosis, with exact binomial 95% confidence intervals. Within each AI system, paired full-size and cropped classifications of the same radiographs were compared using two-sided exact McNemar tests. Because human observers evaluated only full-size images, direct human–AI comparisons were restricted to the full-size condition; cropped-image results were considered exclusively as a within-AI analysis of the effect of image framing. Given the different number and dependency structure of observations between human and AI conditions, no pooled omnibus comparison across all evaluator/model conditions was performed.

Inter-observer agreement within each human observer group was assessed using Fleiss’ kappa with 95% confidence intervals. All tests were two-sided, with *p* < 0.05 indicating statistical significance. Analyses were performed using Stata/SE version 19.5 (StataCorp LLC, College Station, TX, USA).

## 3. Results

All 50 selected cases were included. Considering the entire images, overall accuracy was highest among postgraduate students (84.9%), followed by undergraduate students (78.7%). Gemini achieved 72.0% accuracy with full-size images whereas ChatGPT achieved 68.0% (Table 1). The analysis of the two AI software on cropped images provided markedly different outputs as Gemini showed 82.0% accuracy, while ChatGPT achieved 50.0% accuracy (Table 1).

**Figure 1:**
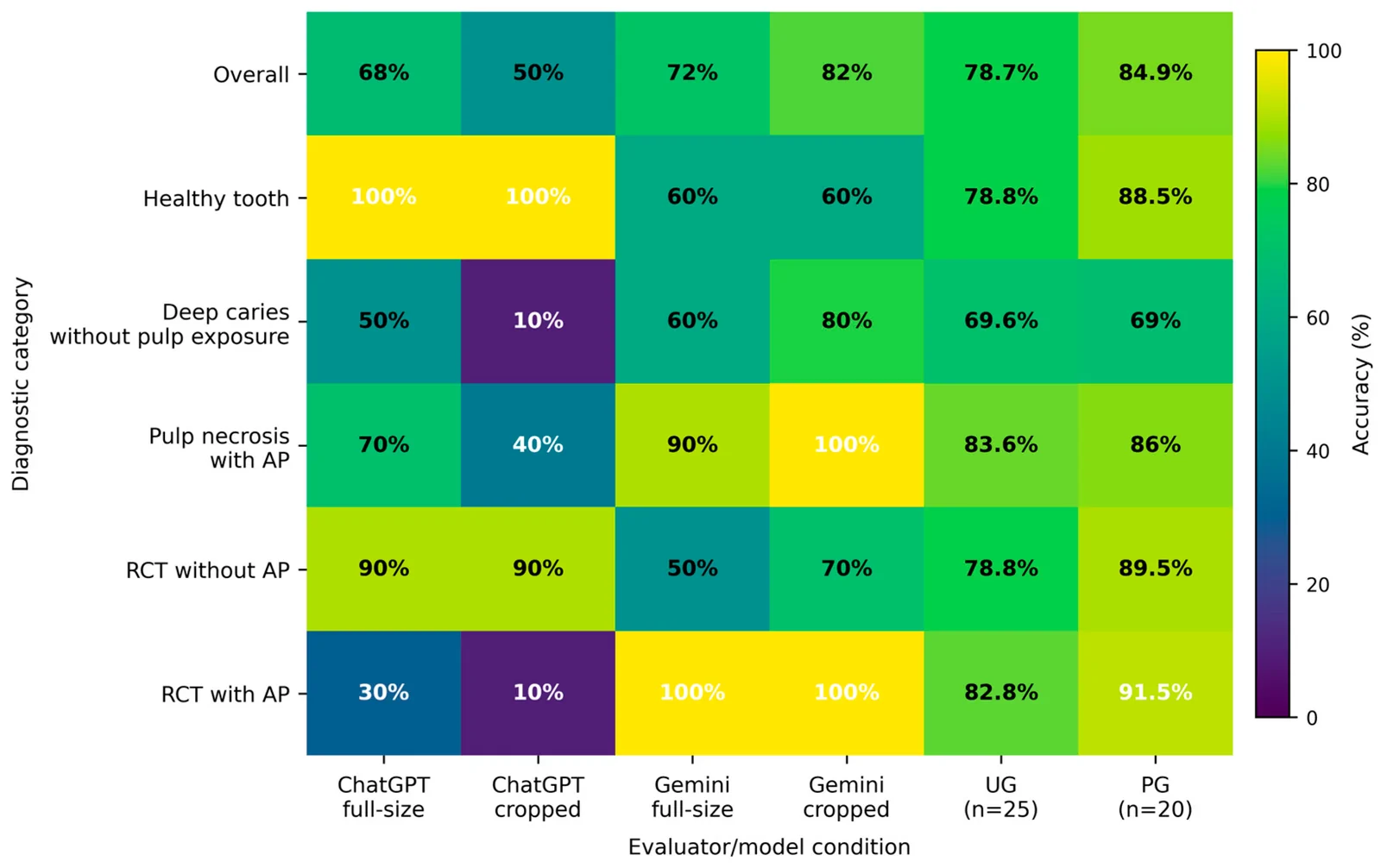
Diagnostic accuracy by evaluator/model condition, image format, and diagnostic category. AI accuracy is shown separately for full-size and cropped periapical radiographs; human accuracy is aggregated by observer group. AP, apical periodontitis; PG, postgraduate students; RCT, root canal treatment; UG, undergraduate students. Human accuracy values are pooled across repeated observer responses (1250 undergraduate and 1000 postgraduate classifications), whereas each AI condition is based on 50 single-pass classifications. Cropped AI results are presented only as a within-model image-format analysis.

Performance was strongly category-dependent. ChatGPT correctly classified all healthy teeth in both formats and 90% of root canal-treated teeth without apical periodontitis, but classified only 30% of full-size images and 10% of cropped root canal-treated teeth with apical periodontitis. Gemini correctly classified all cropped untreated teeth with apical periodontitis and all root canal-treated teeth with apical periodontitis in both formats. Human accuracy was lowest for carious lesions without radiographic pulp exposure (69.6% for undergraduate and 69.0% for postgraduate students). The category-specific pattern is visualized in Figure 1.

Cropping reduced ChatGPT accuracy from 68.0% to 50.0% (exact McNemar *p* = 0.0117), whereas the increase in Gemini accuracy from 72.0% to 82.0% was not statistically significant (*p* = 0.3018).

Agreement with the reference standard ranged from 50.0% to 82.0% across the AI conditions (Table 2). Inter-observer agreement was substantial in both human groups and was higher among postgraduate than undergraduate students (Fleiss’ κ = 0.731 vs. 0.627; Table 3).

Mixed-effects logistic regression accounting for repeated evaluations by observers and clustering by radiographic case showed a significant observer-group-by-diagnostic-category interaction (Wald χ^2^(4) = 10.22, *p* = 0.037). Postgraduate students had higher odds of correct classification than undergraduate students for healthy teeth (OR = 2.28; 95% CI, 1.19–4.38; *p* = 0.013), root canal-treated teeth without apical periodontitis (OR = 2.52; 95% CI, 1.30–4.88; *p* = 0.006), and root canal-treated teeth with apical periodontitis (OR = 2.53; 95% CI, 1.24–5.20; *p* = 0.011), whereas no significant differences were observed for caries without pulp exposure or pulp necrosis with apical periodontitis.

## 4. Discussion

The principal finding was that diagnostic performance varied substantially by evaluator, AI system, image format, and diagnostic category. Postgraduate students achieved the highest overall accuracy, followed by undergraduate students. Among AI systems, Gemini was more accurate and consistent overall, particularly with cropped images. ChatGPT performed well for healthy teeth and root canal-treated teeth without apical periodontitis but performed poorly for post-treatment apical periodontitis. These results do not support treating general-purpose multimodal AI systems as interchangeable diagnostic tools.

Cropping radiographs produced opposite effects in the two systems: Gemini accuracy increased by 10 percentage points, whereas ChatGPT accuracy decreased by 18 points. Focusing on the target tooth may reduce distracting information, but it can also remove adjacent tooth anatomy and periapical tissues.

The present findings match recent endodontic studies while also illustrating the importance of task design. Durmazpinar and Ekmekci reported 91.4% accuracy for ChatGPT-4o when clinical histories, radiographs, and clinical photographs were presented together [15]. Kurt and Şimşek found 79.6% accuracy for ChatGPT-4o and 71.6% for Gemini on multiple-choice questions concerning pulpal and periapical diseases [16]. By contrast, Almutairi et al. reported only 54.0–63.2% accuracy for ChatGPT-5 on radiographs of endodontically treated teeth, with particularly low sensitivity for voids and periapical lesions [18]. This aligns with ChatGPT performance for root canal-treated teeth with apical periodontitis in the present study. Differences among studies are therefore likely to reflect not only model capability but also the availability of clinical context, image type, diagnostic categories and prompt design [17,21,22].

Human performance was more stable across diagnostic categories. Postgraduate students achieved the highest overall accuracy. The lowest human accuracy occurred for carious lesions without radiographic pulp exposure. Estimating caries depth and its relation to the pulp on a two-dimensional image can be difficult, and the absence of symptoms, sensibility testing, or direct clinical examination further limits certainty [9,10,23].

Some clarifications are necessary in relation to the findings for caries without pulp exposure and pulp necrosis with apical periodontitis. For caries without pulp exposure, the absence of radiographic pulp exposure does not establish a healthy pulp or exclude histological pulpal inflammation. For pulp necrosis with apical periodontitis, radiographic findings compatible with apical periodontitis in an untreated tooth do not independently establish pulp necrosis. Accuracy for these diagnostic conditions therefore represents agreement with the clinical reference diagnoses based on the radiographic information provided to the evaluators, rather than independent confirmation of pulpal status. The detection of apical periodontitis in root canal-treated teeth is clinically important because it may influence monitoring, nonsurgical retreatment, apical surgery, extraction, or replacement [12,24]. Dedicated deep-learning systems have shown promising performance for periapical radiolucencies [7,8], but general-purpose multimodal systems are not trained or validated for a single dental imaging task. The current results support a role as supervised educational or second-opinion tools rather than as autonomous diagnostic systems. Structured AI descriptions may encourage systematic examination of coronal integrity, root fillings, periodontal ligament space, lamina dura, and periapical tissues, but a clinician must verify the final interpretation [4,15,16,18,25].

The use of a single standardized prompt improved experimental consistency but does not establish prompt robustness. Multimodal AI systems may respond differently to changes in wording, task framing, output constraints, or instructions regarding unavailable clinical information. The instruction not to infer clinical information may also interact differently with each model’s pretrained priors and internal processing [22].

Several limitations should be considered. Only 50 radiographs were included, with 10 selected cases per category; this balanced design does not reproduce disease prevalence or the full spectrum of clinical scenario.

The reference standard was based on available clinical and radiographic documentation rather than histopathology or three-dimensional imaging. Previous investigations have also highlighted the limitations of radiographic assessment in endodontics, showing that residual root canal filling materials may remain undetected even when both periapical radiographs and CBCT are used [26]. Human observers were assessed without clinical information, and the AI systems were evaluated in a single prompt-based workflow. The study did not test repeated sessions, response stability, alternative prompts, image quality levels, or additional model versions. Therefore, AI results should be interpreted as first-response diagnostic performance rather than as estimates of inter-session reliability or reproducibility. Because proprietary systems change over time, these results represent the evaluated versions and cannot be generalized to later updates. Finally, although PowerPoint was configured to use High fidelity with image compression disabled, some lossy recompression or resampling can occur during export. Some differences between full-size and cropped AI performances could be attributed to these changes.

## 5. Conclusions

General-purpose multimodal AI systems showed heterogeneous performance in endodontic radiographic classification. Accuracy depended on the system, image format, and diagnostic category. Postgraduate students achieved the highest overall accuracy, while Gemini approached human performance in selected categories and ChatGPT showed marked weakness in post-treatment apical periodontitis, especially with cropped images. These findings reflect radiographic classification under controlled conditions and should not be interpreted as equivalent to comprehensive endodontic diagnosis, which requires integration of radiographic findings with clinical examination and patient-related information. These systems may support education and supervised radiographic review, but they should not replace comprehensive clinical assessment or trained professional judgment.

## Figures and Tables

**Table 1:** Diagnostic accuracy by evaluator/model condition, image format, and diagnostic category.

	AI Software				Human Observer	
Diagnostic Category	ChatGPT Full-Size	ChatGPT Cropped	Gemini Full-Size	Gemini Cropped	Undergraduate Cohort	Postgraduate Cohort
Healthy tooth	10/10 (100.0)	10/10 (100.0)	6/10 (60.0)	6/10 (60.0)	197/250 (78.8)	177/200 (88.5)
Caries without pulp exposure	5/10 (50.0)	1/10 (10.0)	6/10 (60.0)	8/10 (80.0)	174/250 (69.6)	138/200 (69.0)
Pulp necrosis with AP	7/10 (70.0)	4/10 (40.0)	9/10 (90.0)	10/10 (100.0)	209/250 (83.6)	172/200 (86.0)
RCT without AP	9/10 (90.0)	9/10 (90.0)	5/10 (50.0)	7/10 (70.0)	197/250 (78.8)	179/200 (89.5)
RCT with AP	3/10 (30.0)	1/10 (10.0)	10/10 (100.0)	10/10 (100.0)	207/250 (82.8)	183/200 (91.5)
Overall	34/50 (68.0)	25/50 (50.0)	36/50 (72.0)	41/50 (82.0)	984/1250 (78.7)	849/1000 (84.9)

Values are correct classifications/total classifications (%). **AP**, apical periodontitis; **RCT**, root canal treatment.

**Table 2:** Agreement between each AI Software and the reference standard.

AI Software	Agreement with Reference Standard	95% CI
ChatGPT, full-size	34/50 (68.0%)	53.3–80.5%
ChatGPT, cropped	25/50 (50.0%)	35.5–64.5%
Gemini, full-size	36/50 (72.0%)	57.5–83.8%
Gemini, cropped	41/50 (82.0%)	68.6–91.4%

**Table 3:** Inter-observer agreement within the human observer groups.

Observer Group	Observers	Cases	Fleiss’ κ	95% CI
Undergraduate students	25	50	0.627	0.558–0.685
Postgraduate students	20	50	0.731	0.667–0.786

## Data Availability

The original contributions presented in this study are included in the article. Further inquiries can be directed to the corresponding author.

## Appendix A. Standardized AI Prompt

For each radiograph, the following standardized prompt was used:

Please analyze the target tooth #[insert tooth number according to FDI notation] in this periapical radiograph.

Base your assessment exclusively on the radiographic findings visible in the image. Do not infer or assume any clinical information that is not provided, including symptoms, pulp sensibility test results, percussion or palpation findings, periodontal probing, or patient history.

Evaluate the following radiographic features:

1. coronal integrity and presence of carious radiolucencies;
2. depth of any carious lesion and its relationship with the pulp chamber;
3. presence or absence of previous root canal treatment;
4. periodontal ligament space and lamina dura;
5. presence or absence of periapical radiolucency;
6. periapical status of the target tooth;
7. alveolar bone crest and surrounding supporting tissues.

Based on these observations, assign the target tooth to one and only one of the following diagnostic categories:

(A) Healthy tooth
(B) Tooth with a deep carious lesion without radiographic pulp involvement and without apical periodontitis
(C) Untreated tooth with apical periodontitis compatible with pulp necrosis
(D) Root canal-treated tooth without apical periodontitis
(E) Root canal-treated tooth with apical periodontitis

If the diagnosis is uncertain, select the category that appears most likely based on the radiographic findings. Do not create additional diagnostic categories.

Please provide the final answer in the following format:

Final diagnosis: [A/B/C/D/E]
